# Supplementary material for: Marker Assisted Introgression of Resistance Genes and Phenotypic Evaluation Enabled Identification of Durable and Broad-Spectrum Blast Resistance in Elite Rice Cultivar, CO 51
Source: Genes (Basel). 2023 Mar 15;14(3):719. doi: 10.3390/genes14030719 (PMC10048046; doi:10.3390/genes14030719)
Supplement: Supplementary file 1 [file genes-14-00719-s001.zip › genes-2091643-supplementary.pdf]

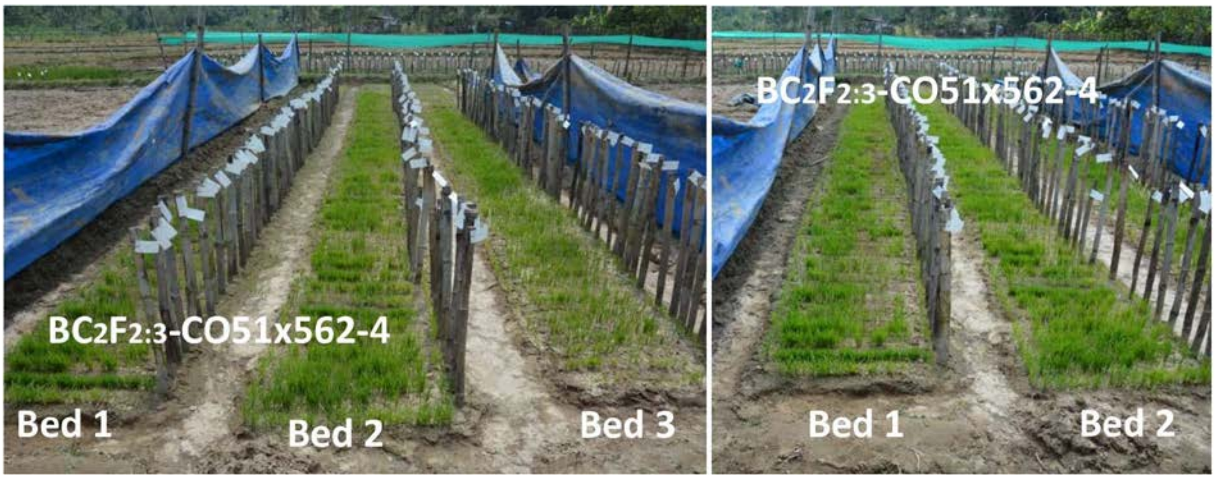

Figure S1. A uniform nursery bed (UNB) raised at Hybrid Rice Evaluation Centre, Gudalur, Tamil Nadu, India for screening of blasé disease in rice.

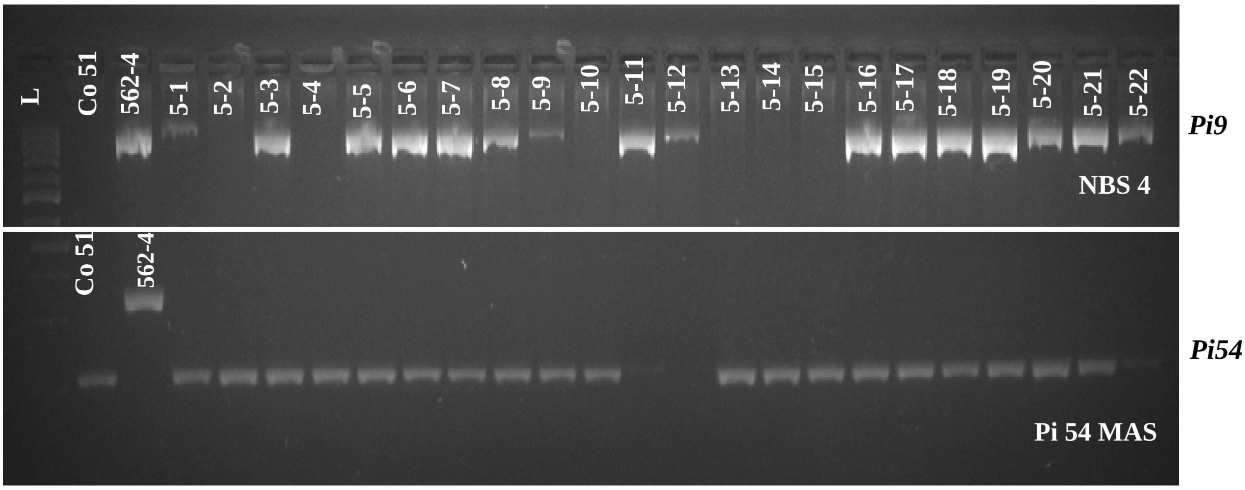

Figure S2. Foreground selection of 22 BC_2F_2 plants using functional markers NBS4 and Pi54MAS linked to *Pi9* and *Pi54*, respectively. CO 51 (Recurrent parent), 562-4 (Donor Parent).

Table S1. List of 55 SSR markers used for background selection.

Sl.No	Locus	Chr.No.	Sl.No	Locus	Chr.No.
1	RM490	1	29	RM276	6
2	RM8051	1	30	RM508	6
3	RM594	1	31	RM400	6
4	RM312	1	32	RM585	6
5	RM283	1	33	RM30	6
6	RM431	1	34	RM4608	6
7	RM8077	1	35	RM527	6
8	RM221	2	36	RM11	7
9	RM207	2	37	RM6728	7
10	RM208	2	38	RM447	8
11	RM213	2	39	RM310	8
12	RM424	2	40	RM152	8
13	RM6	2	41	RM219	9
14	RM3202	3	42	RM239	10
15	RM520	3	43	RM216	10
16	RM426	3	44	RM271	10
17	RM3843	4	45	RM286	11
18	RM3276	4	46	RM287	11
19	RM261	4	47	RM5857	11
20	RM7313	4	48	RM552	11
21	RM185	4	49	RM254	11
22	RM6172	4	50	RM27230	11
23	RM5749	4	51	RM235	12
24	RM241	4	52	RM1302	12
25	RM274	5	53	RM247	12
26	RM289	5	54	RM512	12
27	RM164	5	55	RM270	12
28	RM510	6			
